# TRIC-A Facilitates Sarcoplasmic Reticulum–Mitochondrial Ca^2+^ Signaling Crosstalk in Cardiomyocytes

**DOI:** 10.3390/cells14201579

**Published:** 2025-10-11

**Authors:** Ang Li, Xinyu Zhou, Ki Ho Park, Jianxun Yi, Xuejun Li, Jae-Kyun Ko, Yuchen Chen, Miyuki Nishi, Daiju Yamazaki, Hiroshi Takeshima, Jingsong Zhou, Jianjie Ma

**Affiliations:** 1 Department of Kinesiology, College of Nursing and Health Innovation, University of Texas at Arlington, Arlington, TX 76010, USA; ang.li3@uta.edu (A.L.);; 2 Department of Surgery, Division of Surgical Sciences, University of Virginia, Charlottesville, VA 22903, USA; ywx5wp@virginia.edu (X.Z.); uuh2qa@virginia.edu (K.H.P.);; 3 Department of Pharmacology, Kyoto University, Kyoto 606-8501, Japan; 4 Division of Pharmacology, National Institute of Health Sciences, Tokyo 158-8501, Japan

**Keywords:** mitochondrial Ca^2+^ homeostasis, reactive oxygen species, Ca^2+^ biosensor, SOICR, TMEM38A

## Abstract

TRIC-A is an intracellular cation channel enriched in excitable tissues that is recently identified as a key modulator of sarcoplasmic reticulum (SR) Ca^2+^ homeostasis through direct interaction with type 2 ryanodine receptors (RyR_2_). Given the intimate anatomical and functional coupling between the SR and mitochondria, we investigated whether TRIC-A contributes to SR–mitochondrial crosstalk under cardiac stress conditions. Using a transverse aortic constriction (TAC) model, we found that TRIC-A^−/−^ mice developed more severe cardiac hypertrophy, underwent maladaptive remodeling, and activated apoptotic pathways compared with wild-type littermates. At the cellular level, TRIC-A-deficient cardiomyocytes were more susceptible to H_2_O_2_-induced mitochondrial injury and displayed abnormal mitochondrial morphology. Live-cell imaging revealed exaggerated mitochondrial Ca^2+^ uptake during caffeine stimulation and increased propensity for store-overload-induced Ca^2+^ release (SOICR). Complementary studies in HEK293 cells expressing RyR_2_ demonstrated that exogenous TRIC-A expression attenuates RyR_2_-mediated mitochondrial Ca^2+^ overload, preserves respiratory function, and suppresses superoxide generation. Together, these findings identify TRIC-A as a critical regulator of SR–mitochondrial Ca^2+^ signaling. By constraining mitochondrial Ca^2+^ influx and limiting oxidative stress, TRIC-A safeguards cardiomyocytes against SOICR-driven injury and confers protection against pressure overload-induced cardiac dysfunction.

## 1. Introduction

Mitochondria occupy more than 30% of the cytoplasmic volume in adult cardiomyocytes and generate over 90% of the adenosine triphosphate (ATP) required for contraction, ion transport, and other energy-demanding processes [1,2,3,4]. These organelles are also the predominant intracellular source of reactive oxygen species (ROS) and play a pivotal role in initiating programmed cell death [3,5,6,7,8,9,10]. Structural and biochemical abnormalities of mitochondria are consistently observed across diverse cardiomyopathies, underscoring their vulnerability in cardiac disease [2,11,12].

Calcium ions within the mitochondrial matrix ([Ca^2+^]_mito_) serve as a pivotal regulator of ATP synthesis by activating multiple enzymes related to the tricarboxylic acid (TCA) cycle and oxidative phosphorylation [13,14,15]. However, pathological [Ca^2+^]_mito_ overload, which is often coupled with ROS-induced oxidative stress, promotes sustained opening of the mitochondrial permeability-transition pore (mPTP). Prolonged mPTP opening leads to mitochondrial matrix swelling, dissipation of the inner-mitochondrial-membrane (IMM) potential, ATP depletion, rupture of the outer-mitochondrial membrane (OMM), and eventual cardiomyocyte death [8,9,16,17,18]. Persistent mPTP activation has been implicated in ischemia–reperfusion injury, and interventions that constrain pore opening have been used to attenuate infarct size and adverse ventricular remodeling [8,9,16,17,18].

Sarcoplasmic/endoplasmic reticulum (SR/ER) is the principal intracellular Ca^2+^ reservoir in cardiomyocytes, where Ca^2+^ release through ryanodine receptor 2 (RyR_2_) governs excitation–contraction coupling and strongly influences mitochondrial Ca^2+^ uptake [19,20,21,22,23,24]. In cardiomyocytes, extracellular [Ca^2+^] is ~1–2 mM, while resting cytosolic [Ca^2+^] is ~100 nM, with beat-to-beat values rising into the µM range. The SR lumen maintains sub-millimolar to millimolar levels of Ca^2+^, and mitochondrial matrix [Ca^2+^] is ~100–200 nM at rest, with transient increases tuned to cytosolic oscillations. These gradients provide the driving force for SR–mitochondrial Ca^2+^ transfer, linking rapid cytosolic signals to mitochondrial function [19]. RyR_2_-mediated Ca^2+^ release is electrogenic, generating a transient negative charge inside the SR lumen. To balance this, trimeric intracellular cation (TRIC) channels, which are K^+^-permeable channels located in the SR/ER, provide counter-ion flux that stabilizes SR membrane potential during Ca^2+^ release [25,26,27,28,29,30], with K^+^ serving as the predominant intracellular monovalent cation. Mammals express two isoforms of TRIC channels: TRIC-B, which is broadly expressed, and TRIC-A, which is enriched in excitable tissues such as skeletal muscle, smooth muscle, and the heart [25].

Ablation of TRIC-A causes altered SR Ca^2+^ signaling and tissue-specific phenotypes. In skeletal muscle, TRIC-A ablation results in reduced Ca^2+^ spark frequency, SR Ca^2+^ overload, and fatigue-induced “mechanical alternans” [31]. In vascular smooth muscle cells (VSMCs), TRIC-A ablation-induced SR Ca^2+^ overload enhances inositol 1,4,5-trisphosphate receptor (IP_3_R)-mediated Ca^2+^ transients and VSMC contraction, resulting in hypertension [32]. In cardiomyocytes, the absence of TRIC-A suppresses spontaneous sparks but exaggerates caffeine-evoked release due to SR luminal Ca^2+^ overload [33,34]. Similar phenotypes are observed in embryonic cardiomyocytes from TRIC-A/TRIC-B double-knockout mice, which are embryonically lethal, further underscoring the essential role of TRIC channels in SR Ca^2+^ handling [25].

TRIC-A has also been demonstrated to physically interact with RyR_2_ through its carboxyl-terminal tail domain (CTT) and hence significantly elevates the probability of RyR_2_ opening [33,35]. Thus, TRIC-A plays an essential role in keeping SR Ca^2+^ levels in check, preventing the occurrence of store overload-induced Ca^2+^ release (SOICR), an excitation-independent trigger of cytosolic Ca^2+^ waves that can cause cardiac arrhythmia, and potentially mitochondria damage [36].

Although TRIC-A is established as a regulator of SR Ca^2+^ release, its role in shaping SR–mitochondrial Ca^2+^ crosstalk and the consequent impact on mitochondrial integrity and cardiomyocyte survival remains unclear; we hypothesize that TRIC-A safeguards the heart under stress by constraining RyR_2_-driven mitochondrial Ca^2+^ overload and oxidative injury. In this study, we investigate how TRIC-A modulates mitochondrial Ca^2+^ homeostasis and function in cardiomyocytes under physiological and pathological conditions. By integrating in vivo, cellular, and heterologous expression models, we demonstrate that TRIC-A is essential for constraining RyR_2_-driven mitochondrial Ca^2+^ loading, thereby mitigating oxidative stress, preserving mitochondrial integrity, and protecting the heart from pressure overload-induced remodeling.

## 2. Materials and Methods

### 2.1. Cardiomyocyte Isolation from Adult Mice

TRIC-A-knockout (TRIC-A^−/−^) mice used in this study have been previously described [25]. All animal procedures were conducted in accordance with the Institutional Animal Care and Use Committee (IACUC) guidelines at The Ohio State University and the University of Virginia. Ventricular cardiomyocytes were isolated from adult TRIC-A^−/−^ and wild-type (WT) littermate mice (10–12 weeks, both sexes). Hearts were rapidly excised and perfused via a Langendorff apparatus at 37 °C. Enzymatic digestion was performed by perfusing Tyrode’s solution containing 1 mg/mL collagenase (Type II, 300 U/mg; Worthington, Lakewood, NJ, USA) and 0.1 mg/mL protease (Type XIV, Sigma-Aldrich, St. Louis, MO, USA) for 6 min. Following digestion, ventricles were gently dissociated mechanically to release individual cardiomyocytes, which were used for imaging and functional assays within 3 h. The Tyrode’s solution contained (in mM) 136 NaCl, 5.4 KCl, 0.33 NaH_2_PO_4_, 1.0 MgCl_2_, 10 glucose, and 10 HEPES (pH 7.4).

### 2.2. Transverse Aortic Constriction (TAC) Surgery and Histological Analysis

TAC surgery was performed under continuous isoflurane anesthesia, with body temperature maintained using a heating pad. Analgesia was provided by subcutaneous buprenorphine administration. The thoracic area was shaved and disinfected with alternating betadine and 70% ethanol washes. The surgical field was covered with a sterile Press’n Seal wrap, leaving the incision site exposed. Mice were intubated with a 22G or 24G angiocatheter and mechanically ventilated (Small Animal Ventilator, Model 687, Harvard Apparatus, Holliston, MA, USA) at 80–90 breaths/min with a tidal volume of 0.2–0.3 mL.

A 1 cm left thoracotomy was performed in the upper mid-thorax, followed by blunt dissection of the pectoralis and intercostal muscles. The ribs were retracted, and the left lung and thymus were gently displaced to visualize the transverse aorta posterior to the thymus. Surrounding fat and connective tissue were carefully removed to avoid altering aortic diameter. A 7–0 nylon suture was placed around the aorta, and a pre-sterilized blunt-end 25G, 26G, or 27G needle was positioned alongside it. The suture was tied snugly around the aorta and needle and secured with a double knot, and the needle was removed. Muscles and skin were closed in layers using absorbable sutures. Mice were removed from ventilation and allowed to recover on a heating pad.

Four weeks post-TAC, hearts from TRIC-A^−/−^ and WT mice were harvested, fixed in 10% formalin in PBS, and embedded in paraffin. Serial 4 μm sections were prepared and stained with Hematoxylin and Eosin or Masson’s trichrome for histological analysis of cardiac hypertrophy and fibrosis.

### 2.3. Transmission Electron Microscopy (TEM)

Two weeks post-TAC, left ventricular tissue from TRIC-A^−/−^ and WT mice was prepared for TEM analysis. Tissue samples were fixed in 3% paraformaldehyde and 2.5% glutaraldehyde in a 0.1 M cacodylate buffer (pH 7.4), followed by post-fixation in 1% osmium tetroxide (OsO_4_) in a 0.1 M cacodylate buffer. Samples were then dehydrated and embedded in epoxy resin, and ultra-thin sections were cut using an ultramicrotome. Sections were stained with uranyl acetate and lead citrate before imaging with a transmission electron microscope (JEM-1010, JEOL, Tokyo, Japan) to assess the mitochondrial ultrastructure. Mitochondrial injury was quantified from TEM images using injured mitochondria percentages and the Flameng score (0–4) system [37], where 0 = a normal mitochondrion (intact cristae and dense matrix); 1 = minimal injury (mild cristae loosening); 2 = moderate injury (swelling and partial cristae loss); 3 = severe injury (severe swelling, major cristae disruption, and partial membrane rupture); and 4 = very severe (lysis/rupture of the outer membrane and loss of the matrix).

### 2.4. Ca^2+^ Spark and Wave Measurements in Cardiomyocytes

Intracellular Ca^2+^ sparks and waves in intact adult ventricular cardiomyocytes (4–8 months old) were recorded using a Zeiss LSM 780 confocal microscope equipped with a 40×/1.4 NA oil immersion objective [33] for enhanced light collection and resolution. Cardiomyocytes were loaded with Fluo-4 AM (2 μM) and X-Rhod-1 AM (2 μM) and subjected to field stimulation at 0.5 Hz for 20 s in Tyrode’s solution containing (in mM) 1.8 Ca^2+^, 130 NaCl, 5.6 KCl, 1 MgCl_2_, 11 glucose, and 10 HEPES (pH 7.4). Spontaneous Ca^2+^ sparks were recorded following stimulation at room temperature (24–26 °C). Line-scan images of Fluo-4 fluorescence were acquired at 2 ms per line using the Galvano scan mode of a Nikon A1R confocal microscope. Quantitative analysis and characterization of Ca^2+^ sparks were performed using the SparkMaster plugin for ImageJ (1.54p) [38].

### 2.5. Plasmid Construction

The mouse TRIC-A full-length coding sequence was cloned into EBFP2-N1 (Addgene 54595) between the HindIII and PstI sites to generate TRIC-A–EBFP (enhanced blue fluorescent protein) using the following primers: tttaagcttatggacctgatgtcagcgc and tttctgcagatccgctttcttggtcttcttctt. The mitochondrial-targeted red fluorescent Ca^2+^ sensor 4mt-jRCaMP1b was generated by inserting the jRCaMP1b sequence (Addgene 63136) into a pcDNA-based 4mt construct between the NotI and EcoRI sites using the following primers: tgcggccgcggatctcgcaacaatggtcgac and ggttttgaattcctacttcgctgtcatcatttgtac.

### 2.6. Static and Time-Lapse Imaging of Cells

HEK293 cells with tetracycline-inducible RyR_2_ expression (HEK-tet-RyR_2_) were cultured in DMEM supplemented with 10% FBS and 1% penicillin/streptomycin at 37 °C and 5% CO_2_ [33]. TRIC-A-EBFP, EBFP, 4mt-YC3.6, or 4mt-jRCaMP1b plasmids were transfected using Lipofectamine 3000 per the manufacturer’s instructions. Subcellular localization of fluorescent proteins was verified in fixed cells (4% paraformaldehyde) via staining with PicoGreen (1:400; Thermo Fisher, Waltham, MA, USA), the TRIC-A rabbit antiserum (1:500), or the TOM20 rabbit polyclonal antibody (1:500; 11802-1-AP, Proteintech, Rosemont, IL, USA).

For live-cell imaging, RyR_2_ expression was induced with tetracycline (0.1 μg/mL) 18 h post-transfection, and imaging was performed 18–22 h later. Cytosolic and mitochondrial Ca^2+^ dynamics were monitored after loading cells with Fluo-4 AM (2.5 μM) for 40 min at 37 °C, followed by five washes with Ca^2+^-free Ringer’s solution. Time-lapse recordings were acquired at 0.5 Hz for 8 min at room temperature. Static imaging of 4mt-YC3.6-transfected cells was performed after five washes with Ringer’s solution containing 2.5 mM Ca^2+^. All images were captured using a Leica TCS SP8 confocal microscope with a 63×/1.4 NA oil immersion objective.

Colocalization analysis, line-scan kymography, and ROI intensity measurements were performed in ImageJ. Pseudocolor heatmaps of 4mt-YC3.6 YFP/CFP ratios were generated in MATLAB. (2025a) Absolute mitochondrial Ca^2+^ concentrations were calculated using the corrected ratio method [39], where [Ca^2+^] = K_d_ ((R − R_min_)/(R_max_ − R))^1/n^. The parameters K_d_ = 250 nM, R_min_ = 1.4, R_max_ = 9.3, and n = 1.7 were from previous reports [40]. Curve fitting and calculation of full width at half maximum (FWHM) were performed in MATLAB. Statistical analyses (Student’s t-test or the Wilcoxon rank-sum test) and box-and-dot plotting were performed in R.

### 2.7. Seahorse XF Mito Stress Test

Cultured HEK-tet-RyR_2_ cells were transfected with EBFP or TRIC-A-EBFP 18 h before being reseeded onto 24-well assay plates at 2.5–3 × 10^4^ cells/well with or without tetracycline induction (0.1 µg/mL, 18 h). Cells were washed twice in a pre-warmed serum-free XF assay medium (DMEM buffered with HEPES, pH 7.4) supplemented with 1 mM sodium pyruvate, 1 mM glutamate, and 10 mM glucose. The cells were incubated in a 37 °C non-CO_2_ incubator for 1.5 h before measurement. For the Mito Stress test, oligomycin (2 μM), FCCP (1–2 μM), and rotenone and antimycin A (0.5 μM) were sequentially administered. Oxygen consumption rate (OCR) and extracellular acidification rate (ECAR) were measured simultaneously by the Seahorse XFe 24 analyzer (Agilent, Santa Clara, CA, USA). Basal respiration was calculated by subtracting the non-mitochondrial OCR (OCR4, averaged from 3 measurements) from the OCR without treatment (OCR1, averaged from 3 measurements) and then divided by OCR4 for normalization. ATP-linked respiration was calculated by subtracting the OCR after oligomycin treatment (OCR2, averaged from 3 measurements) from the OCR without treatment (OCR1) and dividing by OCR4 for normalization. Maximum respiration was calculated by subtracting OCR4 from the OCR after FCCP treatment (OCR3, the maximum of the 3 measurement results, as our cell line cannot maintain this OCR even when we increase the final FCCP concentration to 2 µM, the highest recommended concentration). Due to the previous report of an increased coefficient of variation after normalization with cell number/well, we used OCR instead of cell number for normalization, as recommended [41]. Yet we did image the cell nucleus and estimated the cell density per well to identify outlier wells to exclude them from further analysis, as described below: after the Mito Stress test, the cells were fixed with 4% paraformaldehyde for 15 min, briefly washed with PBS containing 30 mM glycine, permeabilized with PBS containing 0.1% Triton-X and Tween 20 for 30 min, and stained overnight with methyl green (4 µg/mL) at 4 °C to highlight the nucleus. Cell nuclei and TRIC-EBFP (or EBFP) signals were imaged under an epifluorescent microscope the next day. A total of 3–4 images were captured per well. The areas of all the nuclei were measured after thresholding and dividing the area of the whole image. Those wells showed large differences in cell density compared to neighboring wells, and they were excluded from the statistical analysis of the normalized respiration rate.

### 2.8. MitoSOX Red and ROS Brite 670 Staining

Mitochondrial and cytosolic ROS were assessed using MitoSOX Red (5 mM stock in DMSO; M36008, Thermo Fisher, Waltham, MA, USA) and ROS Brite 670 (10 mM stock; 16002, AAT Bioquest, Pleasanton, CA, USA), applied at a 1:2000 dilution in culture media for 15 min at 37 °C. Cells were washed five times with Ringer’s solution containing 2.5 mM Ca^2+^ and imaged using a Leica TCS SP8 confocal microscope with a 63×/1.4 NA oil immersion lens.

### 2.9. Statistical Analysis

Data are presented as the mean ± SD unless otherwise specified. The sample size (n) for each experiment is indicated in the figure legends. Normality of the data distribution was assessed, and the statistical test was chosen accordingly. For datasets that were normally distributed, a two-tailed unpaired Student’s t-test was used for comparisons between two groups. For datasets that did not meet normality assumptions, the non-parametric Wilcoxon rank-sum test was applied. For multiple group comparisons, one-way ANOVA with Tukey’s post hoc test was used where appropriate. For time-course or decay kinetics (e.g., TMRE), nonlinear regression fitting was performed, and differences between groups were evaluated using extra sum-of-squares F tests. For Seahorse assays, oxygen consumption rates were normalized to non-mitochondrial respiration, and outlier wells were excluded based on cell density quantification, as described. All tests were two-tailed, and *p* < 0.05 was considered statistically significant. Analyses were performed using GraphPad Prism (10.6.0) and R (4.5.0).

## 3. Results

### 3.1. TRIC-A Protects Against TAC-Induced Cardiomyopathy and Mitochondrial Damage

**Figure 1:**
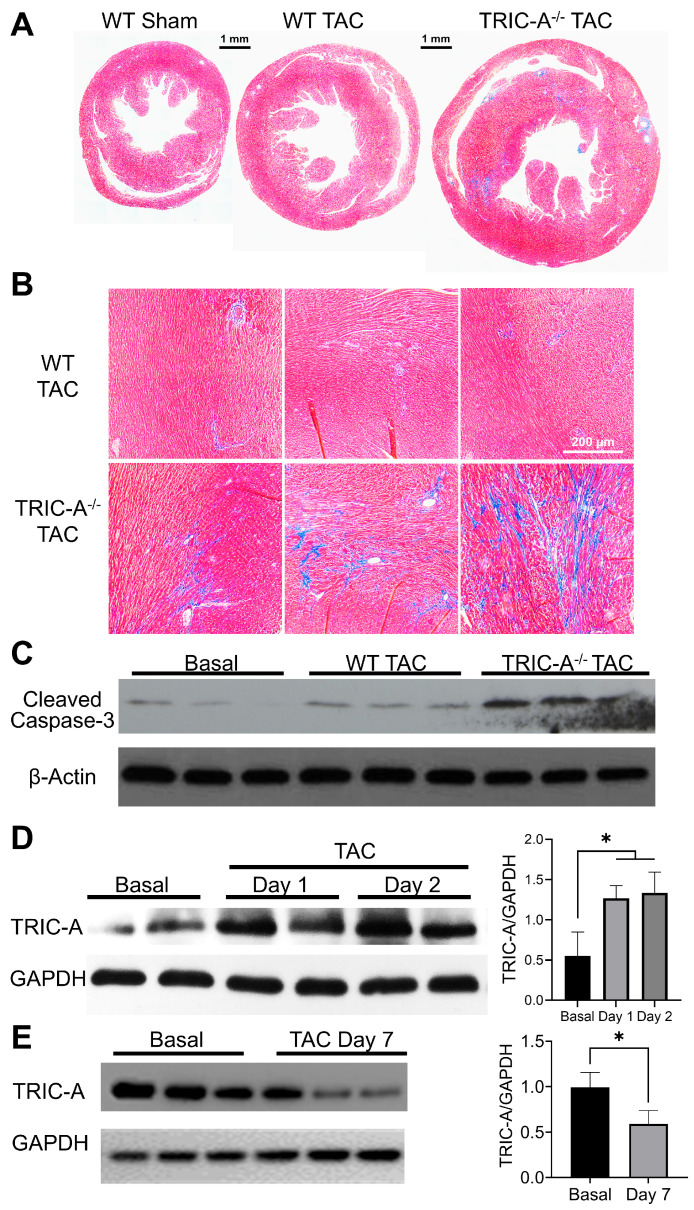
TAC induced more pronounced hypertrophy and fibrosis in TRIC-A^−/−^ hearts. (**A**). Histology of the hearts after 8 weeks of TAC-induced stress. Note the exacerbated hypertrophy phenotype of TRIC-A^−/−^ hearts. (**B**). Masson’s trichrome staining shows increased fibrosis after TAC in TRIC-A^−/−^ hearts. (**C**). TRIC-A^−/−^ hearts exhibit enhanced elevation of cleaved caspase-3 after TAC surgery (*n* = 3). (**D**). The Western blot demonstrates acute induction of TRIC-A in WT hearts after TAC surgery. (*n* = 2, comparing Basal and TAC, * *p* < 0.05, Student’s *t*-test). (**E**). The Western blot demonstrates TRIC-A in WT hearts at Day 7 after TAC surgery. (*n* = 3, * *p* < 0.05, Student’s *t*-test).

To determine whether TRIC-A influences cardiac adaptation to pressure overload, we subjected wild-type (WT) and TRIC-A^−/−^ mice to transverse aortic constriction (TAC), a well-established model of pathological hypertrophy and heart failure [42,43]. Eight weeks after TAC, TRIC-A^−/−^ mice exhibited more severe cardiac remodeling than WT littermates. Histological analysis revealed exaggerated cardiac hypertrophy and markedly increased ventricular interstitial fibrosis in TRIC-A^−/−^ hearts (Figure 1A,B). At the molecular level, cleaved caspase-3, a hallmark of the intrinsic, mitochondria-dependent apoptotic pathway [44,45,46], was significantly elevated in TRIC-A^−/−^ hearts (Figure 1C), suggesting preferential activation of upstream mitochondrial apoptotic signaling. Notably, in WT hearts, TAC induced a rapid and transient increase in TRIC-A protein expression (~2-fold at 1–2 days post-surgery; Figure 1D), consistent with an adaptive response to acute hemodynamic stress. By contrast, TRIC-A levels were markedly reduced by Day 7 after TAC (Figure 1E), indicating that the initial compensatory upregulation is only transient. The subsequent decline in TRIC-A expression may reduce the ability of cardiomyocytes to buffer SR Ca^2+^ release, thereby predisposing the heart to maladaptive remodeling under persistent pressure overload, consistent with the pathological phenotypes of hypertrophy and fibrosis observed in Figure 1A,B.

**Figure 2:**
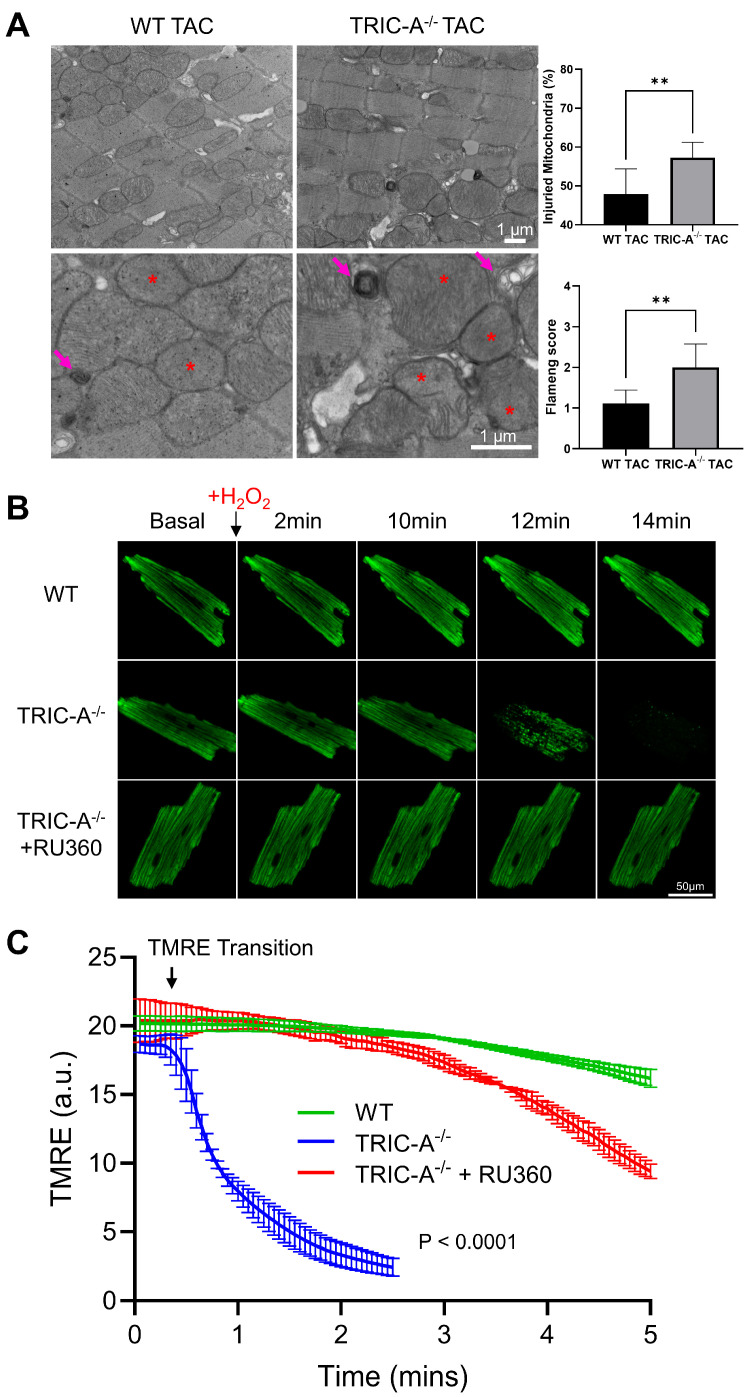
TRIC-A^−/−^ cardiomyocytes exhibit altered mitochondrial morphology and molecular properties. (**A**). Left, EM image of mitochondria of WT and TRIC-A^−/−^ left ventricle tissue after 2 weeks of TAC. Mitochondrial injury is indicated by red stars, and abnormal vacuoles are marked by arrows. Right, quantification of mitochondrial injury (*n* = 3/group, ** *p* < 0.01, Student’s *t*-test). (**B**). Confocal images of cardiomyocytes loaded with TMRE following 1 mM H_2_O_2_ treatment. Cardiomyocytes from TRIC-A^−/−^ mice are more susceptible to H_2_O_2_-induced collapsing of inner-mitochondrial-membrane (IMM) potential. (**C**). TMRE traces showing ΔΨm transitions in WT (green), TRIC-A^−/−^ (blue), and TRIC-A^−/−^ + RU360 (red) mice. Traces were time-aligned to the decay onset before averaging. (*n* = 3/group; decay constants were compared by the extra sum-of-squares *F*-test).

Transmission electron microscopy was used to examine the morphological changes in mitochondria in the left ventricular tissue of mice after TAC. To catch early morphological and biochemical changes, the cardiac tissues were examined 2 weeks after TAC-induced stress. TRIC-A^−/−^ cardiomyocytes displayed a significantly higher incidence of mitochondria with disrupted cristae organization and prominent matrix vacuolization compared with WT controls (Figure 2A).

To probe mitochondria function, we assessed inner-mitochondrial-membrane potential (ΔΨ_m_) in isolated ventricular cardiomyocytes using tetramethyl-rhodamine ethyl ester (TMRE) fluorescence. Following exposure to 1 mM H_2_O_2_, TRIC-A^−/−^ cardiomyocytes showed faster TMRE decay, indicating greater ΔΨ_m_ loss compared with WT cells (Figure 2B). Pretreatment with RU360 (2 µM), a selective inhibitor of the mitochondrial Ca^2+^ uniporter (MCU), partially rescued the H_2_O_2_-induced depolarization in TRIC-A–deficient cells (Figure 2C), demonstrating that their increased susceptibility is dependent on excessive mitochondrial Ca^2+^ influx.

### 3.2. Altered SR–Mitochondrial Ca^2+^ Signaling in TRIC-A^−/−^ Cardiomyocytes After TAC

**Figure 3:**
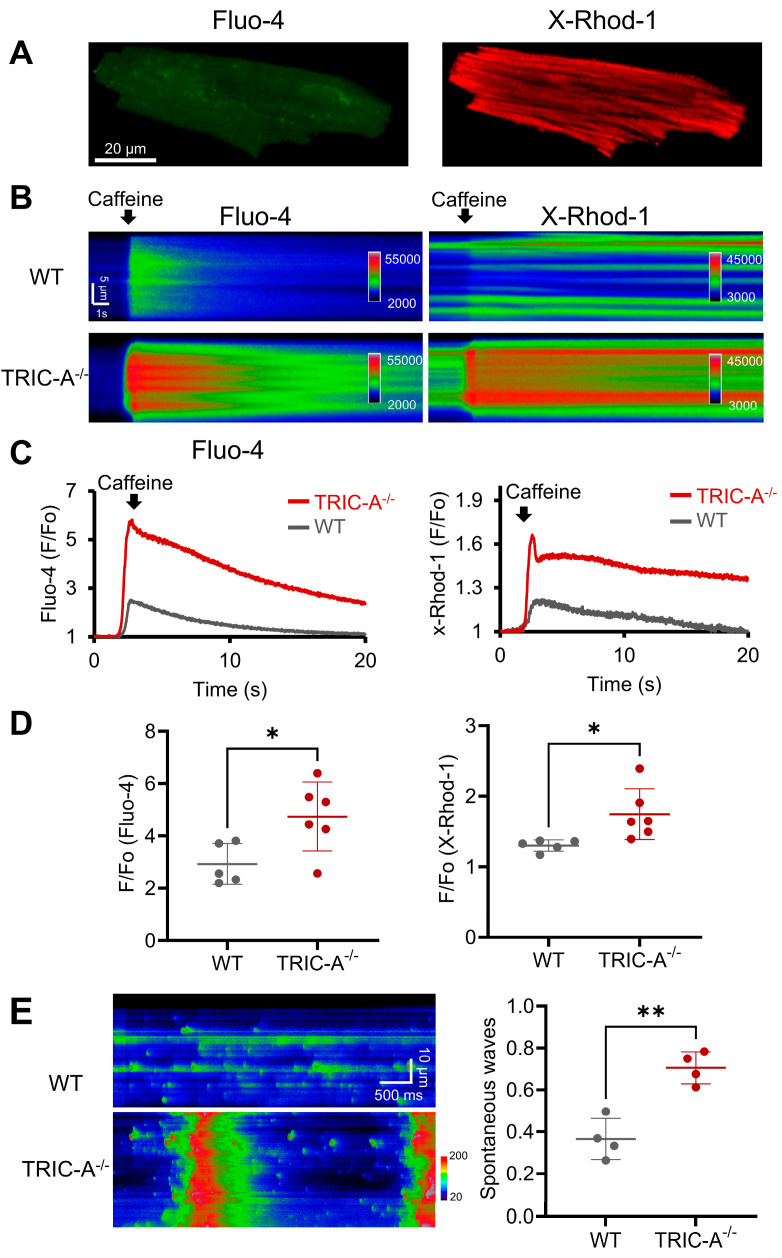
Altered SR and mitochondrial Ca^2+^ signaling in TRIC-A^−/−^ cardiomyocytes after TAC. (**A**). Representative images of isolated cardiomyocytes loaded with Fluo-4 and X-Rhod-1 for monitoring cytosolic and mitochondrial Ca^2+^ dynamics, respectively. (**B**). Representative X-T scan images (pseudo-colored) and corresponding traces. (**C**). Caffeine-induced Ca^2+^ signaling in the cytosol and mitochondria of WT and TRIC-A^−/−^ cardiomyocytes after TAC. (**D**). TRIC-A^−/−^ cardiomyocytes exhibited enhanced cytosolic and mitochondrial Ca^2+^ transients induced by caffeine (*n* = 6 for each group, * *p* < 0.05, Student *t*-test). (**E**). Representative X-T scan images and quantification results demonstrate enhanced spontaneous Ca^2+^ waves in TRIC-A^−/−^ cardiomyocytes (** *p* < 0.01, Student *t*-test).

To directly assess whether SR Ca^2+^ overload underlies the mitochondrial abnormalities observed in TRIC-A-deficient hearts, we analyzed Ca^2+^ dynamics in ventricular myocytes isolated four weeks after TAC. Two fluorescent Ca^2+^ indicators, Fluo-4 AM and X-Rhod-1, were co-loaded to simultaneously monitor cytosolic and mitochondrial Ca^2+^ fluxes, respectively (Figure 3A). Upon caffeine stimulation to activate RyR_2_ channels, TRIC-A^−/−^ myocytes exhibited markedly larger cytosolic Ca^2+^ transients compared with WT controls, accompanied by significantly enhanced mitochondrial Ca^2+^ uptake (Figure 3B–D). These findings indicate that loss of TRIC-A exacerbates RyR_2_-mediated Ca^2+^ release and drives excessive Ca^2+^ transfer into mitochondria.

In addition to evoked release, spontaneous Ca^2+^ activity was also altered. TRIC-A^−/−^ cardiomyocytes displayed a higher frequency of spontaneous Ca^2+^ waves than WT cells (Figure 3E), consistent with a lowered threshold for SOICR. This phenotype mirrors our prior observations in other excitable and non-excitable cell types, including skeletal muscle, vascular smooth muscle, and alveolar type 2 epithelial cells, where TRIC-A deficiency similarly promotes SR/ER Ca^2+^ overload and abnormal Ca^2+^ signaling [31,32,33,34,35].

### 3.3. Exogenous TRIC-A Limits SOICR-Driven Mitochondrial Ca^2+^ Overload in HEK-tet-RyR_2_ Cells

**Figure 4:**
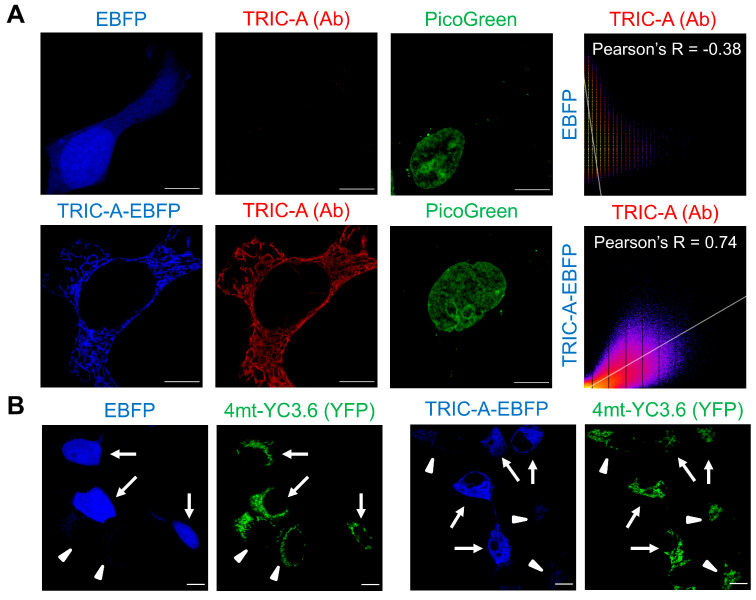
Sparse transfection of HEK-tet-RyR_2_ cells with TRIC-A-EBFP or EBFP alone. (**A**). HEK-tet-RyR_2_ cells transfected with EBFP only or TRIC-A-EBFP and stained with the TRIC-A antibody and the nuclear marker PicoGreen. Pearson’s R reveals the correlation level between two signals (R > 0.5 implies decent colocalization). Scale bar: 10 μm. (**B**). Sparse transfection of 4mt-YC3.6 together with EBFP or TRIC-A-EBFP enables a direct comparison of [Ca^2+^]_mito_ between EBFP-high (or TRIC-A-EBFP) (arrows) and EBFP-low (or TRIC-A-EBFP) (arrowheads) cells within the same view. Scale bars: 10 μm.

To further dissect the role of TRIC-A in regulating SR–mitochondrial Ca^2+^ coupling, we used HEK293 cells with tetracycline-inducible RyR_2_ expression (HEK-tet-RyR_2_) [33,36]. These cells provide a reductionist system to isolate RyR_2_-dependent Ca^2+^ release and evaluate how TRIC-A modulates downstream mitochondrial responses (Figure 4A). Successful overexpression of TRIC-A was verified in cells transfected with TRIC-A–EBFP (enhanced blue fluorescent protein), which displayed strong colocalization between TRIC-A immunostaining and the EBFP signal, whereas EBFP alone showed no overlap. The network-like fluorescence distribution of TRIC-A–EBFP further confirmed its localization to the ER membrane, consistent with its native topology (Figure 4A).

**Figure 5:**
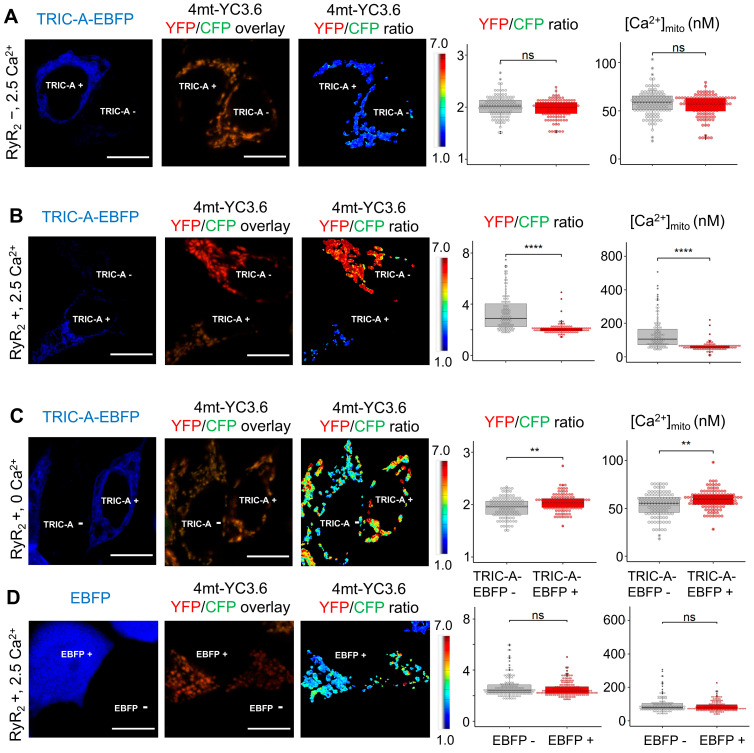
The ratiometric Ca^2+^ sensor 4mt-YC3.6 reveals ameliorated [Ca^2+^]_mito_ overload upon SOICR in HEK-tet-RyR_2_ cells expressing TRIC-A. (**A**–**C**). Representative image and [Ca^2+^]_mito_ quantification in TRIC-A-positive and -negative cells transfected with 4mt-YC3.6 in the absence of RyR_2_ ((**A**), *n* = 119/group), with RyR_2_ and 2.5 mM external Ca^2+^ ((**B**), *n* = 111/group), and with RyR_2_ and 0 mM external Ca^2+^ ((**C**), *n* = 116/group), respectively. (**D**). There is no statistical difference in [Ca^2+^]_mito_ between EBFP-transfected and -un-transfected cells expressing RyR_2_ and exposed to 2.5 mM external Ca^2+^ (*n* = 161/group). For the box-and-dot plot, the box bottom, the median line, and the box top represent the 25th (Q1), 50th (Q2) and 75th percentiles (Q3), respectively. Whisker ends represent Q1 − 1.5*IQR and Q3 + 1.5*IQR, respectively. IQR is the interquartile range (Q3–Q1). ** *p* < 0.01, **** *p* < 0.0001, ns: not significant, Wilcoxon rank-sum test. Scale bars: 10 μm.

To quantify mitochondrial Ca^2+^ dynamics, HEK-tet-RyR_2_ cells were transfected with the ratiometric Ca^2+^ sensor 4mt-YC3.6, whose mitochondrial localization was validated by colocalization with the outer-membrane marker TOM20 (Figure S2). By adjusting the relative transfection ratio of 4mt-YC3.6 with either EBFP or TRIC-A–EBFP, we directly compared [Ca^2+^]_mito_ in TRIC-A-positive versus neighboring TRIC-A-negative cells within the same field (Figure 4B). Under baseline conditions without RyR_2_ induction, [Ca^2+^]_mito_ was indistinguishable between TRIC-A-positive and -negative cells (Figure 5A). However, upon RyR_2_ induction in the presence of 2.5 mM extracellular Ca^2+^, cells lacking TRIC-A-EBFP exhibited robust SOICR and a pronounced elevation in [Ca^2+^]_mito_. In contrast, TRIC-A-overexpressing cells displayed markedly attenuated mitochondrial Ca^2+^ accumulation (Figure 5B). This protective effect is consistent with TRIC-A limiting SR Ca^2+^ overload, likely by enhancing RyR_2_ activation and promoting balanced SR Ca^2+^ release.

Interestingly, in a Ca^2+^-free external solution, TRIC-A–EBFP cells showed a slight increase in basal [Ca^2+^]_mito_ compared with controls, possibly reflecting low-level ER Ca^2+^ leakage, which is associated with TRIC-A activity (Figure 5C). Importantly, no difference in [Ca^2+^]_mito_ was detected between EBFP-only cells and their EBFP-negative neighbors (Figure 5D), confirming that the protective effect arises specifically from TRIC-A rather than EBFP expression.

**Figure 6:**
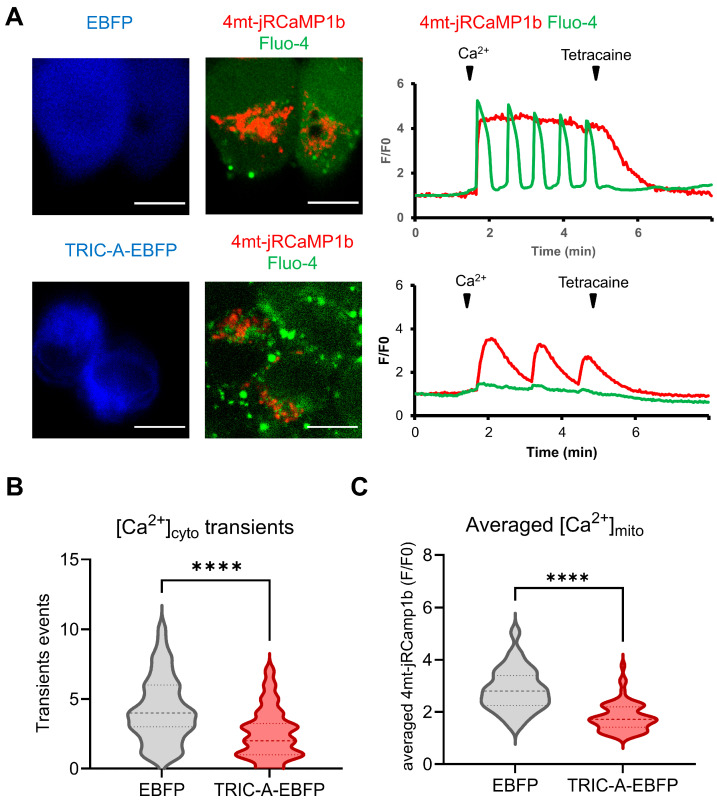
The red-shifted Ca^2+^ sensor 4mt-jRCaMP1b reveals differential [Ca^2+^]_mito_ temporal profiles upon SOICR between TRIC-A positive and -negative cells. (**A**). Cells expressing EBFP alone exhibited frequent [Ca^2+^]_cyto_ oscillations upon administration of Ca^2+^ to the external solution (2 mM), resulting in sustained elevation of [Ca^2+^]_mito_. TRIC-A-EBFP-expressing cells exhibited less frequent and notably smaller [Ca^2+^]_cyto_ transients, leading to either sparser [Ca^2+^]_mito_ elevation or the absence of [Ca^2+^]_mito_ elevation. Tetracaine (RyR_2_ inhibitor) at a concentration of 2 mM was applied 4 min after Ca^2+^ administration to stop [Ca^2+^]_cyto_ oscillations. Scale bars: 10 μm. (**B**). Number of [Ca^2+^]_cyto_ transients within the 4 min period in EBFP- and TRIC-A-EBFP-transfected cells (**** *p* < 0.0001; Student’s *t*-test). (**C**). Average accumulation of [Ca^2+^]_mito_ indicated by 4mt-jRCaMP1b averaged over the 4 min period between the application of external Ca^2+^ and tetracaine in EBFP- (*n* = 59) and TRIC-A-EBFP-transfected (*n* = 66) cells (**** *p* < 0.0001, Student’s *t*-test). For violin plots, the width of the plot represents the data distribution density. The central line indicates the median, and dotted lines show the 25th and 75th percentiles.

We next monitored how mitochondrial Ca^2+^ transients ([Ca^2+^]_mito_) couple to cytosolic Ca^2+^ oscillations ([Ca^2+^]_cyto_) during SOICR. Mitochondria were targeted with the red-shifted Ca^2+^ indicator 4mt-jRCaMP1b [47] (Figure S3), while Fluo-4 AM was used for cytosolic Ca^2+^. In EBFP-only cells, the addition of extracellular Ca^2+^ triggered large and frequent [Ca^2+^]_cyto_ oscillations characteristic of SOICR, which were tightly coupled to sustained elevations of [Ca^2+^]_mito_ (Figure 6A, upper panel). By contrast, TRIC-A-EBFP-expressing cells displayed markedly fewer and smaller cytosolic oscillations, and when oscillations did occur, they produced only modest, pulsatile increases in [Ca^2+^]_mito_ (Figure 6A, lower panel). Consistent with our previous study, quantification of cytosolic Ca^2+^ activity showed that TRIC-A-EBFP-expressing cells exhibited significantly fewer spontaneous [Ca^2+^]_cyto_ transients compared with EBFP controls (Figure 6B). In parallel, average mitochondrial Ca^2+^ accumulation measured with 4mt-jRCaMP1b was markedly reduced in TRIC-A-EBFP-expressing cells (Figure 6C), indicating that TRIC-A ameliorates RyR_2_-driven SOICR-associated sustained elevations of [Ca^2+^]_mito_.

### 3.4. TRIC-A Mitigates Ca^2+^-Stimulated Respiration and Mitochondrial Oxidative Stress

Mitochondrial respiration is tightly coupled to [Ca^2+^]_mito_, which activates key dehydrogenases of the TCA cycle and enhances oxidative phosphorylation. To determine whether TRIC-A regulates Ca^2+^-driven respiratory activity, we performed Seahorse XFe Mito Stress Tests in HEK-tet-RyR_2_ cells with or without TRIC-A expression.

**Figure 7:**
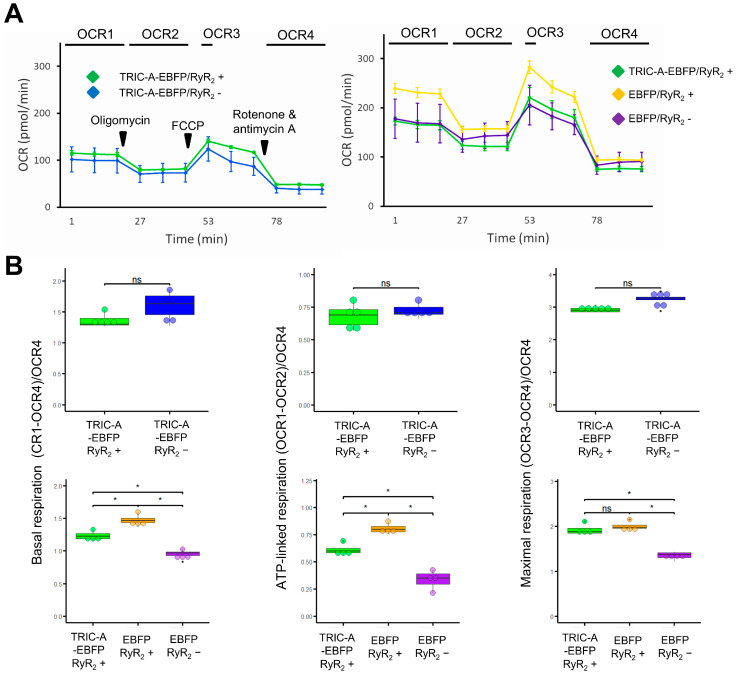
TRIC-A ameliorates the RyR_2_-dependent increase in mitochondrial basal and ATP-linked respiration. (**A**). Oxygen consumption rate (OCR) profile of HEK-tet-RyR_2_ cells measured by the Seahorse XF Mito Stress Test. The left panel compares TRIC-A-EBFP-transfected cells with and without induction of RyR_2_ expression (*n* = 5/group). The right panel compares TRIC-A-EBFP-transfected cells with induction of RyR_2_ expression against EBFP-transfected cells with and without induction of RyR_2_ expression (*n* = 4/group). (**B**). Comparison of basal respiration, ATP-linked respiration, and maximal respiration (all normalized to non-mitochondrial oxygen consumption) between HEK-tet-RyR_2_ under the treatment described above. No statistical differences were found between TRIC-A-EBFP-transfected cells with and without induction of RyR_2_ expression, while EBFP-transfected cells exhibited elevated basal respiration, ATP-linked respiration, and maximal respiration upon expression of RyR_2_. In a side-by-side comparison, the impact of RyR_2_ expression on basal respiration and ATP-linked respiration was less severe in TRIC-A-EBFP-transfected cells. No significant difference in maximal respiration was found (* *p* < 0.05; ns, not significant; Wilcoxon rank-sum test). For the box-and-dot plot, the box bottom, the median line, and the box top represent the 25th (Q1), 50th (Q2) and 75th (Q3) percentiles, respectively. Whisker ends represent Q1 − 1.5*IQR and Q3 + 1.5*IQR, respectively. IQR is the interquartile range (Q3–Q1).

TRIC-A-EBFP-transfected cells exhibited comparable basal, ATP-linked, and maximal respiration (normalized to non-mitochondrial oxygen consumption) with or without RyR_2_ induction, whereas EBFP-transfected cells displayed significantly increased basal, ATP-linked, and maximal respiration upon RyR_2_ induction, consistent with sustained mitochondrial Ca^2+^ entry due to SOICR. In contrast, TRIC-A-EBFP expression blunted the increase in basal and ATP-linked respiration while preserving maximal respiratory capacity in HEK cells in the presence of RyR_2_ (Figure 7A,B; Figure S4).

### 3.5. TRIC-A Ameliorates Mitochondrial Oxidative Stress in HEK-tet-RyR_2_ Cells

**Figure 8:**
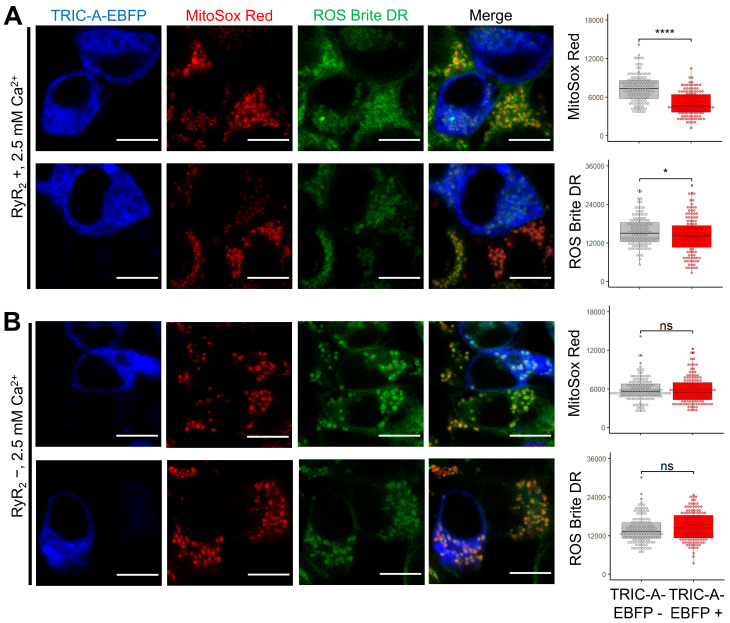
TRIC-A-overexpressed cells exhibit significantly reduced mitochondrial ROS levels. (**A**). HEK-tet-RyR_2_ cells sparsely transfected with TRIC-A-EBFP and stained with MitoSOX Red and ROS Brite 670 to assess oxidative stress in mitochondria and cytosol, respectively. TRIC-A-EBFP-positive cells exhibited significantly reduced mitochondrial superoxide levels compared to TRIC-A-EBFP-negative cells, while the cytosolic ROS levels were only marginally different. (*n* = 128 cells/group; **** *p* < 0.0001; * *p* < 0.05; Wilcoxon rank-sum test). (**B**). No significant differences in oxidative stress in mitochondria or cytosol were detected in TRIC-A-EBFP-positive vs. -negative cells without induction of RyR_2_ expression (*n* = 135 cells/group; NS, not significant; Wilcoxon rank-sum test). For the box-and-dot plot, the box bottom, the median line, and the box top represent the 25th (Q1), 50th (Q2) and 75th (Q3) percentiles, respectively. Whisker ends represent Q1 − 1.5*IQR and Q3 + 1.5*IQR, respectively. IQR is the interquartile range (Q3–Q1).

To determine whether TRIC-A modulates oxidative stress, we assessed mitochondrial and cytosolic ROS levels in HEK-tet-RyR_2_ cells using MitoSOX Red (mitochondrial superoxide) and ROS Brite 670 (whole-cell ROS). In mixed cultures containing both TRIC-A–EBFP-positive and -negative cells, TRIC-A-expressing cells consistently displayed lower MitoSOX fluorescence than their TRIC-A-negative neighbors (Figure 8A), indicating attenuation of mitochondrial superoxide production. In contrast, cytosolic ROS-Brite intensity was only modestly reduced, suggesting that TRIC-A preferentially protects against mitochondrial, rather than global, oxidative stress. Importantly, when RyR_2_ expression was not induced, neither mitochondrial superoxide nor cytosolic ROS levels differed between TRIC-A-EBFP-positive and -negative cells (Figure 8B).

## 4. Discussion

The precise coordination of Ca^2+^ signaling crosstalk between SR and mitochondria is a fundamental determinant of cardiac physiology, governing mitochondrial bioenergetics, ROS production, and programmed cell death [48,49,50]. Direct Ca^2+^ transfer at ER/SR–mitochondrial junctions, particularly through RyR_2_-mediated release, is critical for regulating mitochondrial metabolic adaptation and cardiomyocyte survival [51,52,53]. While TRIC-A has been recognized as a modulator of SR Ca^2+^ homeostasis, its role in linking SR Ca^2+^ release to mitochondrial function remained unexplored [33]. Here, we identify TRIC-A as a pivotal mediator of SR–mitochondrial Ca^2+^ crosstalk, which is important for protecting mitochondrial function and mitigating oxidative stress under conditions of increased cardiac demand.

Interestingly, we observed biphasic regulation of TRIC-A expression following pressure overload: a rapid induction 1–2 days post-TAC followed by a marked reduction by Day 7. This response profile suggests that TRIC-A is mobilized as an early adaptive mechanism to stabilize SR–mitochondrial Ca^2+^ signaling and protect mitochondrial integrity during acute stress. However, its downregulation under persistent overload may compromise this protective capacity, thereby weakening mitochondrial defenses and facilitating maladaptive remodeling. A similar response has been reported for SERCA2a, a central regulator of SR Ca^2+^ reuptake, which shows increased expression during the compensatory phase after the TAC but declines with progression to failure [54]. This finding highlights the importance of TRIC-A in the adaptive response to pressure overload: its transient upregulation supports Ca^2+^ homeostasis in the early phase, whereas its subsequent loss removes a critical safeguard, accelerating the transition from compensated hypertrophy to maladaptive remodeling.

Under basal conditions, SR Ca^2+^ load and RyR_2_ activity are modest, and adaptive or redundant mechanisms likely compensate for TRIC-A deficiency, minimizing overt phenotypes. Under stress, such as pressure overload, accelerated Ca^2+^ cycling increases SR Ca^2+^ accumulation and mitochondrial uptake. In wild-type hearts, TRIC-A provides counter-ion buffering and modulates RyR_2_ gating to prevent pathological store overload-induced Ca^2+^ release (SOICR). TRIC-A deficiency compromises this adaptive response, resulting in uncontrolled cytosolic Ca^2+^ release, mitochondrial Ca^2+^ overload, and ROS accumulation. The ensuing feedback loop destabilizes both RyR_2_ activity and cytosolic Ca^2+^ homeostasis, culminating in mitochondrial depolarization, cytochrome c release, cardiomyocyte death, and fibrotic remodeling.

Under RyR_2_ induction, TRIC-A-positive cells showed lower basal and ATP-linked respiration with preserved maximal capacity, consistent with maintenance of mitochondrial reserves under stress. Lower mitochondrial superoxide levels specifically under RyR2 induction indicate that TRIC-A’s protection from oxidative stress is closely tied to RyR2-mediated Ca^2+^ transfer. At the cellular and organ levels, our findings establish TRIC-A as a nodal regulator that couples SR excitability to mitochondrial metabolic adaptation. Efficient SR-to-mitochondrial Ca^2+^ transfer ensures timely ATP production in high-demand cardiac cells. Dysregulation of this process contributes to the pathogenesis of heart failure, arrhythmia, and stress-induced cardiomyopathy. By extending the functional paradigm of TRIC-A beyond a passive counter-ion channel to an active modulator of organellar communication, our work provides a mechanistic framework linking ion channel regulation, bioenergetics, and cell survival.

Reconstitution studies in HEK-tet-RyR_2_ cells provide further mechanistic insight, taking advantage of the well-established and widely used HEK293-RyR_2_ platform in cardiac Ca^2+^ signaling research [36]. This system enables precise evaluation of RyR_2_-dependent Ca^2+^ signaling and downstream mitochondrial Ca^2+^ responses under defined conditions [55,56,57]. In our experiments, TRIC-A expression attenuates RyR_2_-dependent mitochondrial superoxide production without altering cytosolic ROS, demonstrating a mitochondria-targeted protective function. Critically, this effect is RyR_2_-dependent, highlighting TRIC-A’s role in modulating Ca^2+^ transfer specifically through SR–mitochondrial microdomains. Collectively, these observations support a model in which TRIC-A restrains excessive mitochondrial Ca^2+^ uptake under conditions of SOICR, thereby limiting ROS generation, preserving mitochondrial integrity, and maintaining cardiomyocyte survival.

Recent studies reinforce the importance of balanced SR–mitochondrial crosstalk in heart physiology and disease [58]. SR Ca^2+^ signaling via RyR_2_/SOICR has emerged as a key upstream trigger of mitochondrial Ca^2+^ overload and downstream stress [59]. Work in adult cardiomyocytes highlights SR–mitochondrial microdomains as structured, dynamic sites that must deliver balanced Ca^2+^ flux: too little blunts energetic reserve, whereas excess promotes mitochondrial Ca^2+^ overload and oxidative injury [60]. Within this context, our data identify TRIC-A as a regulator that limits RyR_2_-driven mitochondrial Ca^2+^ loading under stress, reducing ROS while maintaining physiological signaling. These findings position TRIC-A as a microdomain regulator of SR–mitochondrial coupling in cardiac disease.

From a translational perspective, TRIC-A represents a promising therapeutic target. Enhancing TRIC-A activity could strengthen mitochondrial resilience in conditions characterized by Ca^2+^ mishandling and oxidative stress, such as ischemia–reperfusion injury and chronic pressure-overload cardiomyopathy. Conversely, aberrant TRIC-A signaling could promote arrhythmogenic Ca^2+^ waves in cardiomyocytes, emphasizing the context-dependent nature of TRIC-A function and the importance of precision-targeted interventions. Although no agonists of TRIC-A are currently available, the development and screening of small-molecule modulators hold translational potential. In parallel, we are currently developing a MyoAAV-mediated gene delivery approach for the TRIC-A C-terminal peptide, which is aimed at restoring SR–mitochondrial Ca^2+^ handling and improving cardiac function. Looking forward, TRIC-A modulation could also be explored in combination with other interventions, such as inhibition of the mitochondrial Ca^2+^ uniporter (MCU), to further limit Ca^2+^-driven oxidative injury. While these approaches remain at an early stage, they underscore the potential of TRIC-A as a novel entry point for protecting mitochondrial integrity and cardiac function in disease.

Several limitations of the current study should be considered: First, although we primarily relied on murine cardiomyocytes and HEK-tet-RyR_2_ cells, the HEK system is a widely used platform for dissecting RyR_2_ and SOICR mechanisms, but it does not fully capture cardiac physiology. Future studies in human iPSC-derived cardiomyocytes and in vivo stress models will be important to extend our findings. Second, while our work focused on SR–mitochondrial coupling, other Ca^2+^-storing organelles such as lysosomes can contribute to local microdomains, although their influence on mitochondrial uptake in adult cardiomyocytes appears limited. Third, the precise molecular interfaces through which TRIC-A organizes SR–mitochondrial contacts remain unclear and will require high-resolution imaging and proteomic approaches. Finally, although we demonstrate that TRIC-A reduces mitochondrial Ca^2+^ overload and oxidative stress during acute stress, the long-term effects on remodeling, arrhythmogenesis, and metabolism remain unexplored.

In conclusion, our study establishes TRIC-A as a central mediator of SR–mitochondrial Ca^2+^ crosstalk, which is important for maintaining mitochondrial bioenergetics and redox homeostasis in cardiomyocytes. By regulating RyR_2_-dependent Ca^2+^ microdomains, TRIC-A protects against mitochondrial overload, oxidative stress, and cardiomyocyte death. These findings redefine the physiological role of TRIC-A and provide a foundation for exploring its therapeutic potential in cardiac diseases characterized by Ca^2+^ dysregulation and mitochondrial dysfunction.

## Data Availability

The data supporting the findings of this study are available from the corresponding authors upon reasonable request.

## Supplementary Materials

The following supporting information can be downloaded at: https://www.mdpi.com/article/10.3390/cells14201579/s1, Figure S1: Original western blot supporting Figure 1; Figure S2: Validation of mitochondrial localization of the ratiometric Ca^2+^ sensor 4mt-YC3.6; Figure S3: Validation of mitochondrial localization of red-shifted Ca^2+^ sensor 4mt-jRCaMP1b; Figure S4: Assessment of cell density after Seahorse XF Mito Stress Test to exclude outliers.

## Abbreviations

The following abbreviations are used in this manuscript:

ER/SR	Endoplasmic/sarcoplasmic reticulum
CICR	Calcium-induced calcium release
IMM	Inner mitochondrial membrane
SOICR	Store overload-induced calcium release
ECG	Electrocardiogram
RyR	Ryanodine receptor
TRICs	Trimeric intracellular cation channels
CTT	Carboxyl-terminal tail
EBFP	Enhanced blue fluorescent protein
