# Supplementary material for: TRIC-A Facilitates Sarcoplasmic Reticulum–Mitochondrial Ca2+ Signaling Crosstalk in Cardiomyocytes
Source: Cells. 2025 Oct 11;14(20):1579. doi: 10.3390/cells14201579 (PMC12564512; doi:10.3390/cells14201579)
Supplement: Supplementary file 1 [file cells-14-01579-s001.zip › cells-3901170-supplementary.pdf]

Supplementary Figure

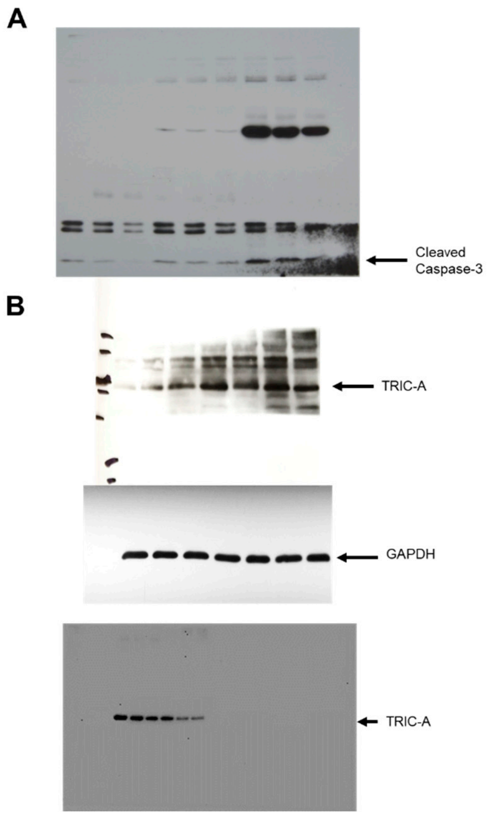

Figure S1. Original western blot supporting Figure 1.

A. Original western blot showing cleaved caspase-3 expression in TRIC-A$^{-/-}$ and WT hearts following TAC surgery. **B.** Original western blot showing TRIC-A protein expression in WT hearts after TAC surgery

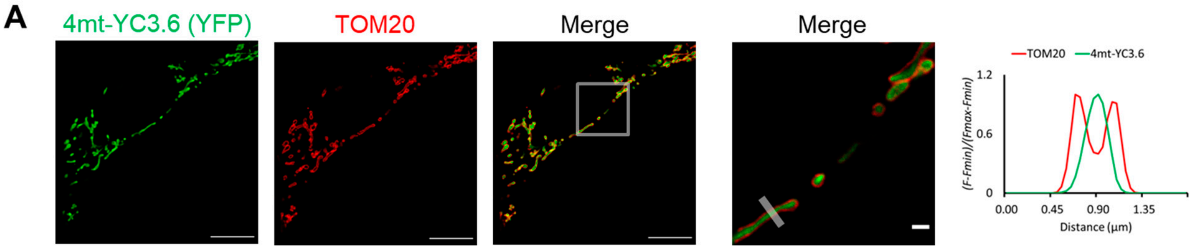

Figure S2. Validation of mitochondrial localization of the ratiometric Ca^{2+} sensor 4mt-YC3.6.

A. Intensity profile comparison of 4mt-YC3.6 fluorescence with the mitochondrial outer-membrane marker TOM20. The boxed region on the left image is enlarged on the right. The white line denotes the area used for intensity profiling. Scale bars: 10 μm (left), 1 μm (right).

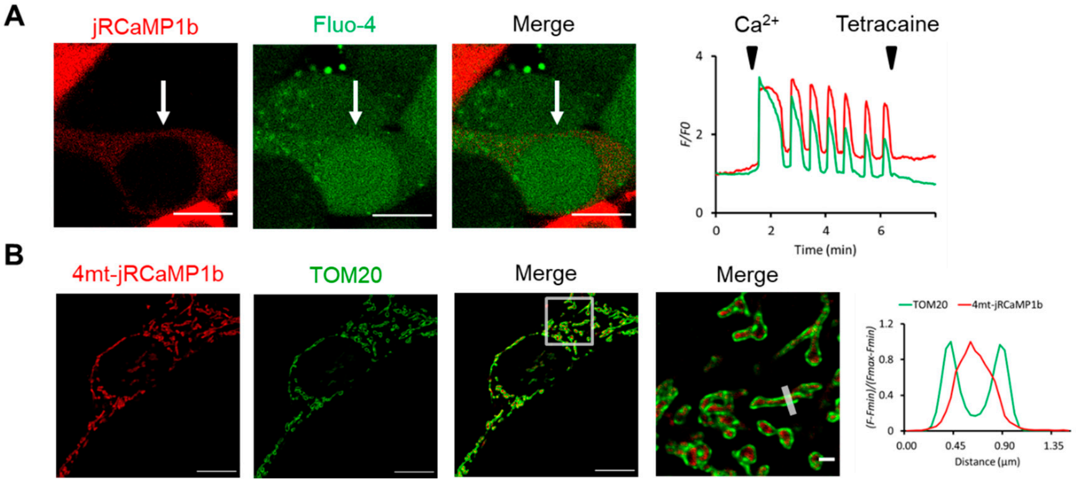

Figure S3. Validation of mitochondrial localization of red-shifted Ca^{2+} sensor 4mt-jRCaMP1b.

A. Representative traces of jRCaMP1b (cytosolic) and Fluo-4 in HEK-tet-RyR_2 cells (arrows, n = 6) underwent sequential treatment of 2 mM Ca^{2+} and tetracaine, respectively (arrowheads). Scale bars: 10 µm**. B.** Intensity profile comparison of 4mt-jRCaMP1b fluorescence with the mitochondrial outer-membrane marker TOM20. The boxed region on the left image is enlarged on the right. The white strip denotes the area used for intensity profiling. Scale bars: 10 µm (left), 1 µm (right).

Figure S4

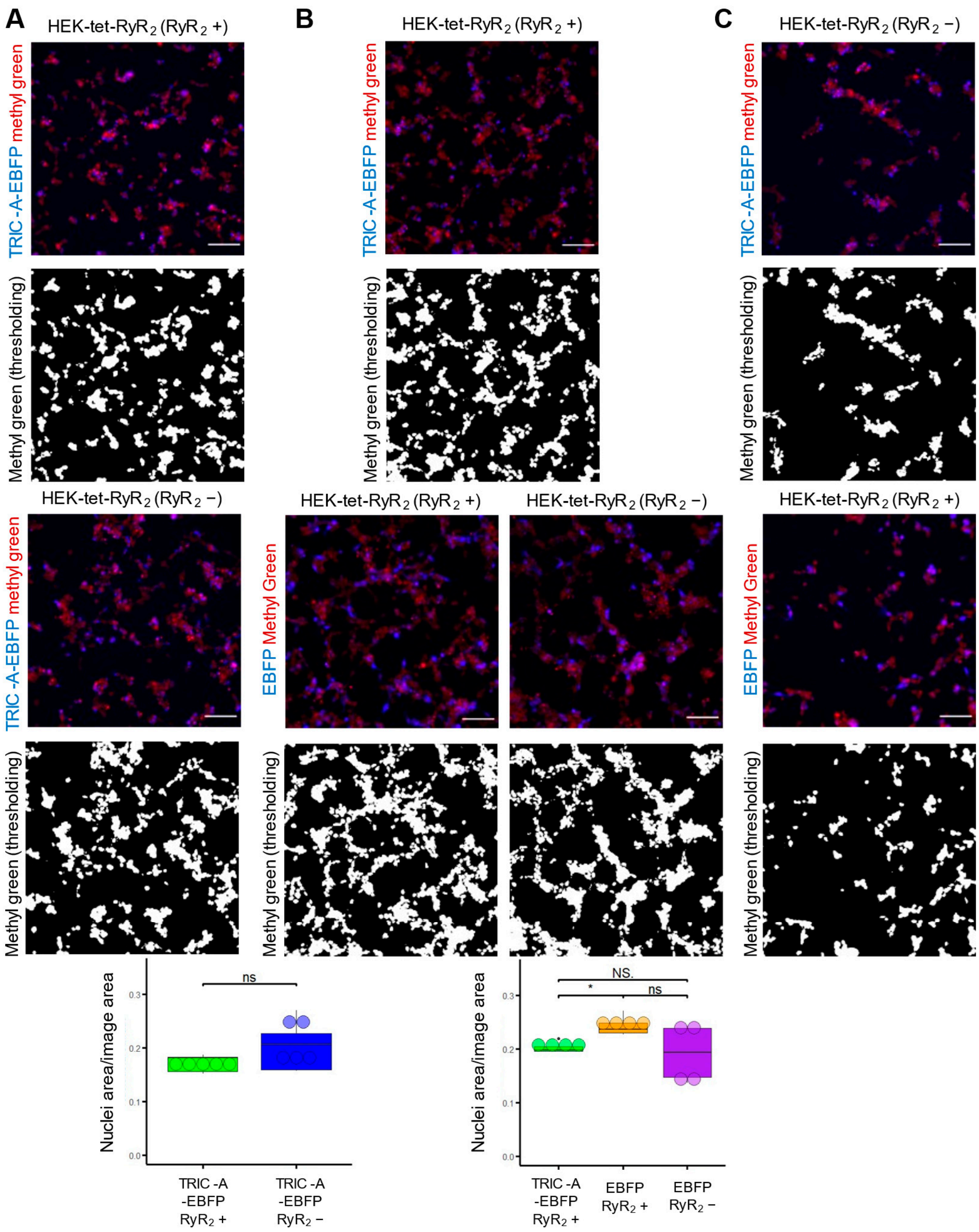

Figure S4. Assessment of cell density after Seahorse XF Mito Stress Test to exclude outliers. A. Representative images of HEK-tet-RyR_2 cells seeded in Seahorse XF24 cell culture microplates after Mito Stress Test in Figure 7. The cells were fixed in 4% paraformaldehyde and stained with Methyl green to highlight nuclei. The lower panels are the same images after thresholding/smoothing on methyl green channel used for quantification. Scale bars, 100 μm. **B.** Quantification of the ratios between total nuclei area and image area (3-4 images captured and averaged per well, each dot represents one well, * $P < 0.05$, ns, not significant, Wilcoxon rank-sum test). For the box and dot plot, the box bottom, median line, and box top represent the 25th (Q1), 50th (Q2) and 75th percentile (Q3), respectively. Whisker ends represent Q1 – 1.5*IQR and Q3 + 1.5*IQR, respectively. IQR is interquartile range (Q3-Q1). **C.** Examples of outlier wells excluded from analysis based on abnormal cell density.
